# Designing a 3D Printed Model of the Skull-Base: A Collaboration Between Clinicians and Industry

**DOI:** 10.1177/23821205221080703

**Published:** 2022-03-07

**Authors:** Youssuf Saleh, Rory Piper, Michael Richard, Sanjeeva Jeyaretna, Thomas Cosker

**Affiliations:** 1 Nuffield Department of Clinical Neurosciences, University of Oxford, UK; 2 Department of Neurosurgery, John Radcliffe Hospital, Oxford University Hospitals, UK; 3 3D Life Prints, Nuffield Orthopaedic Centre, Oxford, United kingdom; 4 Department of Physiology Anatomy and Genetics, University of Oxford, UK

## Abstract

**Introduction:**

The role of three dimensional (3D) printing in neurosurgical education is becoming increasingly common. Notably, 3D printing can simulate complex anatomical pathways that may be difficult to regularly and accurately reproduce in cadavers. One such example is the course of the facial nerve within the temporal bone and its relation to the labyrinth. This can aid pre-surgical planning and minimise surgical complications. Here we aim to develop a novel anatomically accurate model of the skull base which demonstrates key neuro vascular components and the course of the facial nerve within the temporal bone by developing a 3D printed model of the skull-base that can be used for medical education and pre-surgical planning.

**Materials and Methods:**

We utilised a combination of Computed Tomography (CT) and angiography scans to reconstruct the skull base and its vascular contents. Neural components were digitally incorporated under the guidance of the Oxford neurosurgical team and the anatomy department. The model was integrated and printed using polymer jetting.

**Results:**

The model was successfully printed, with all neurovascular components included. Notably we were able to highlight the intra-temporal course of the facial nerve by creating a bony window within the temporal bone.

**Conclusion:**

Through a collaboration with industry and a multidisciplinary team, we were able to reproduce the base of the skull from patient neuro-imaging. Our model is both cost-effective, reproducible and can aid both medical students and neurosurgical trainees in their training/education.

## Introduction

Three-dimensional (3D) printing, also known as additive manufacturing, has developed significantly since its inception.^1,2^ Its applications are now widely used within the medical field, spanning across multiple specialties.^1^ One novel use of 3D printing in healthcare has been the development of anatomical models for surgical education and training.^3,4^ This has been partly driven by a gradual reduction in undergraduate anatomy curricula and access to cadaveric teaching.^5^ Additionally, the use of adjunctive teaching tools in surgical training has become increasingly important following the reduction in training hours stipulated by the European working directive.^6,7^ Moving forward, innovative learning tools are likely to serve as valuable adjuncts alongside cadaveric teaching which is often considered the gold standard method of teaching anatomy. A recent review of best practices in anatomical teaching highlighted 3D printing models as a tool with great promise for teaching anatomy as well as surgical planning.^8^

Three-dimensional printed models can serve as a particularly important learning tool for neurosurgical trainees who operate around intricate and complex neurovascular structures.^9^ In-vivo, anatomical knowledge of these structures is critical to avoid operative complications, which is not gained directly from the traditional two-dimensional images in textbooks and online resources. This can be particularly valuable in skull-base surgery, where operators often drill through the temporal bone to access the deep recesses of the skull base including the cerebello-pontine angle (CPA), infratemporal fossa and clivus.^10,11^ In doing so, damage to important structures such as the cochlea, facial nerve or carotid artery may result in severe disability including hearing loss, facial disfigurement or stroke.^12^ Identifying the complex course of important key structures within the Internal Auditory Canal (IAC) and ‘rock-like’ petrosal part of the temporal bone, such as the facial nerve and/or internal carotid artery, is imperative for improved patient outcomes in this challenging patient group.^13^ Furthermore, the utility of 3D anatomy in these complex cases was identified early on by pioneering surgeons and anatomists with the production of published atlases.^14^ However, they remain limited by the need for 3D projection devices and/or the use of polarised glasses to create the illusion of a 3D image.

We looked to circumvent these issues and develop a true scalable 3D printed model of the skull base. We also chose to recreate the relational anatomy of the brainstem, the cranial nerves and the neurovascular network to the skull base, as it is the clinically most relevant anatomy. Given the complexity of the facial nerve within the temporal bone and its overriding importance, a central feature of this model is the ability to visualise the cochlea, labyrinth and facial nerve within the temporal bone.

The aim of this project was to develop a highly anatomically accurate model that would be of use for students of anatomy at various levels of training. We expect that it will be of utility for both junior medical students with little direct experience of the relevant neuroanatomy to surgeons with a greater level of experience. Here we outline the steps required to produce this prototype, followed by a discussion of its advantages relative to current available options. Finally, we consider the limitations and future directions of 3D printing in this field.

## Materials and Methods

Our model was created in collaboration between the University of Oxford and 3D LifePrints, a 3D technology company with a particular interest in applications to the medical sector. We aimed to balance the level of detail within the model against the educational value delivered to the learner. The model was designed to tend to the learning requirements of medical students and neurosurgical trainees learning to navigate the anterior circulation within the skull base. The model was funded by the Nuffield Orthopaedic Center (Oxford University Hospitals) and consisted of a hemi-section of the skull base and its contents which included:

1. The five bones of the skull base (frontal, temporal, sphenoid, ethmoid and occipital) and their foramina. Our model was designed as a sagittal hemi section to maximise the visual trajectory of the cranial nerves and arterial supply within the skull base. Further, we exposed the temporal bone to showcase the entire course of the facial nerve within the internal acoustic canal. These decisions meant that the cribiform foraminae, and the hiatus of the greater and lesser petrosal nerves were not visible. As we prioritised the needs of surgical trainees with an interest in skull base surgery, these concessions were offset by the benefit of having uninterrupted views of the cranial nerves and anterior circulation.
2. The cochlea and labyrinth
3. The arterial supply including: Internal carotid artery (ICA), middle cerebral artery (MCA), anterior cerebral artery (ACA), posterior cerebral and superior cerebellar artery. The Internal jugular vein, superior sagittal sinus and straight sinus were also included within the model to highlight the key components of the venous system.
4. The brainstem.
5. The cranial nerves (I-XII). All twelve cranial nerves were modelled.

### Neuroimaging

**Figure 1.**
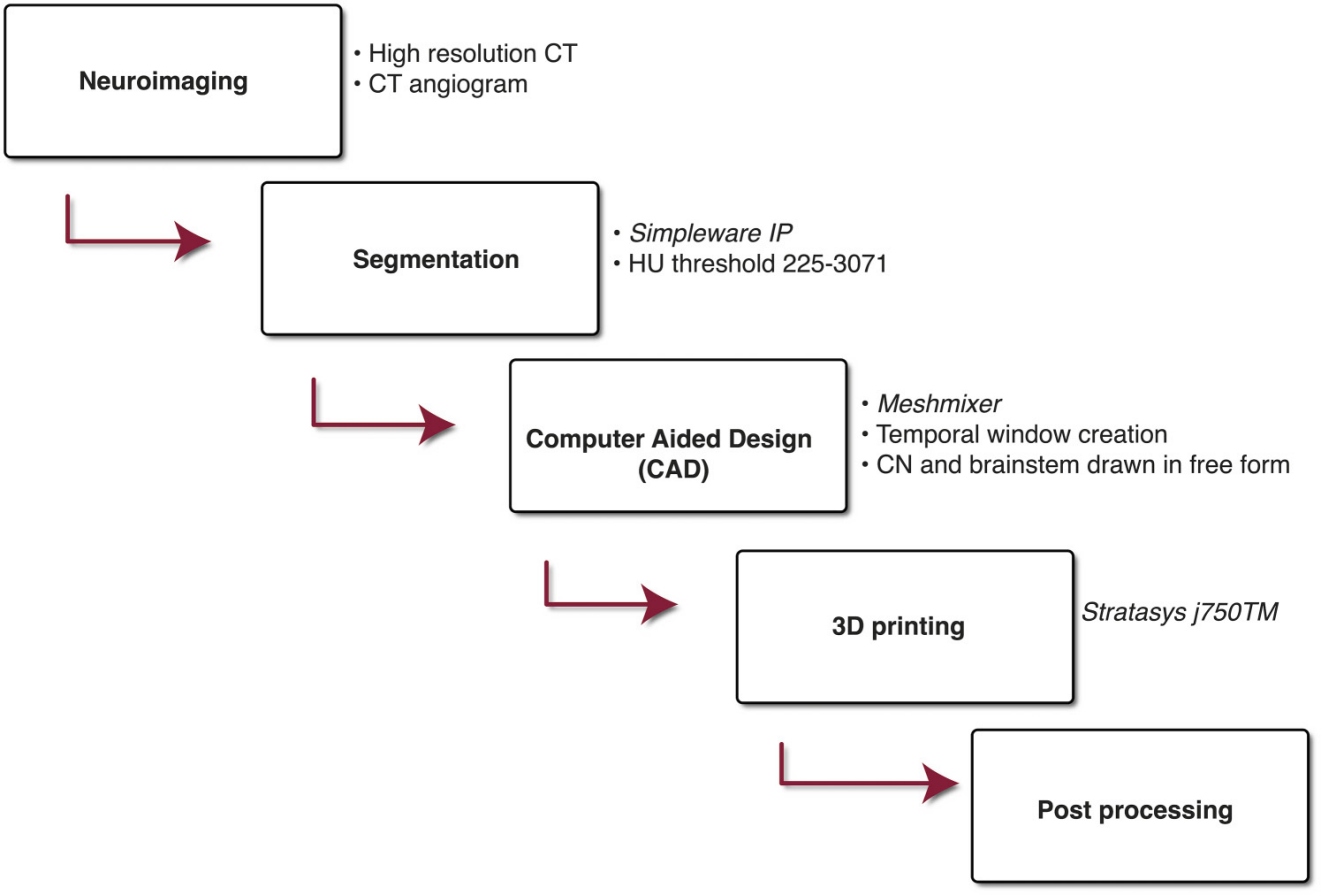
Project workflow: A summary of the stages required to create our model.

To improve anatomical accuracy and reconstruct in-vivo similarity, components 1-3 were recreated from patient neuroimaging sequences in DICOM format. These included high-resolution computer tomography (CT) and CT angiogram images. Images were obtained with a 64-slice CT (GE LightSpeed VCT, John Radcliffe Hospital, Oxford, United Kingdom). The standard emergency scanning protocol was used with the following parameters: 120 kV, Collimation width 0.625 mm, convolution kernel for bone, pitch 0.53, revolution time 0.5 s, image matrix 512 × 512, and pixel size 0.48 mm. We used two separate subjects’ scans for the bony and vascular representations respectively. These images were anonymised to preserve patient confidentiality. The brain stem and all 12 cranial nerves were entirely constructed through a collaboration of anatomy artists and a team of clinicians including neurologists and neurosurgeons. An overview of the project workflow can be found in Figure 1.

### Registration and Segmentation

**Figure 2.**
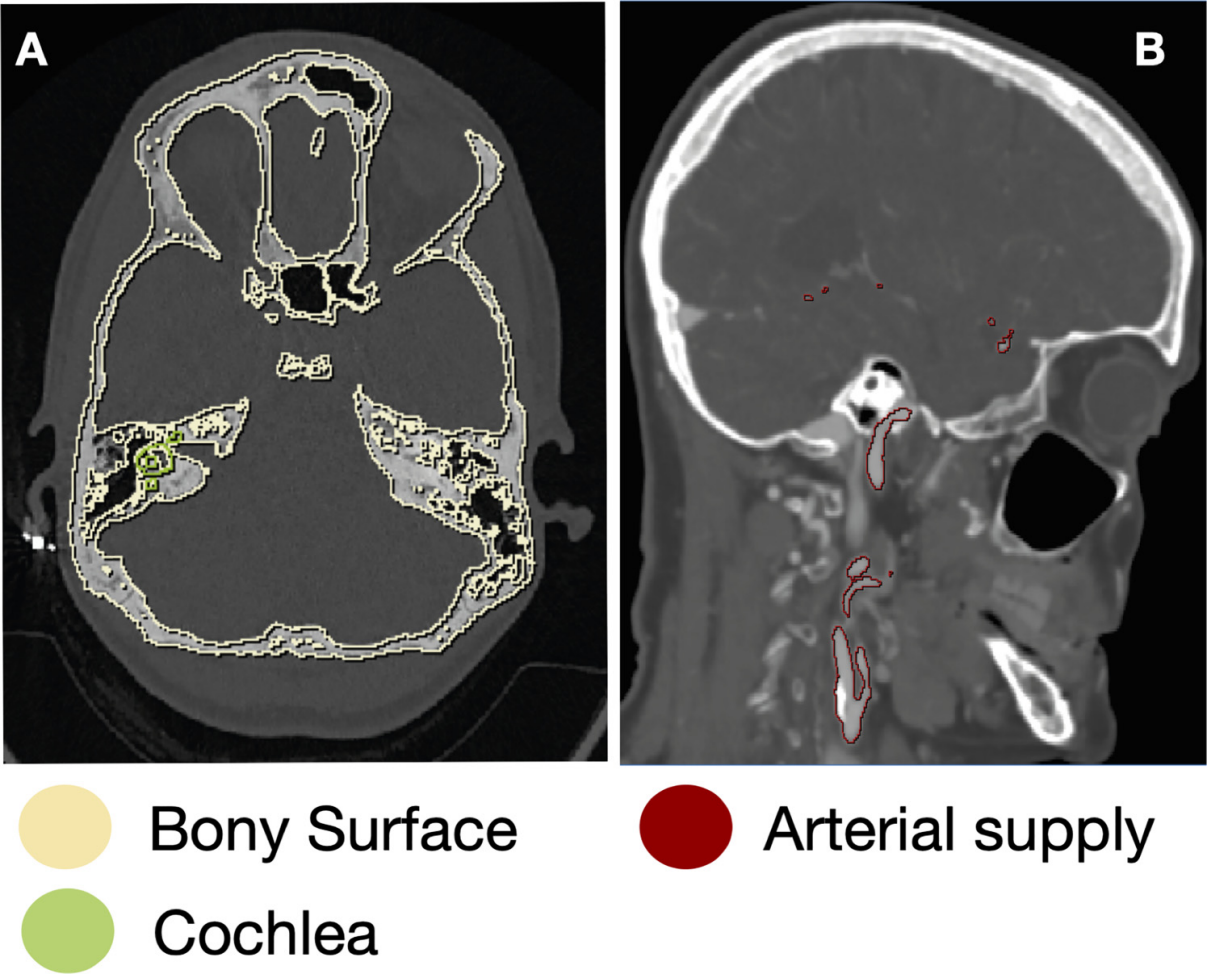
Tissue segmentation: semiautomated segmentation of the bony surface, (A), and the neurovascular supply (B).

**Figure 3.**
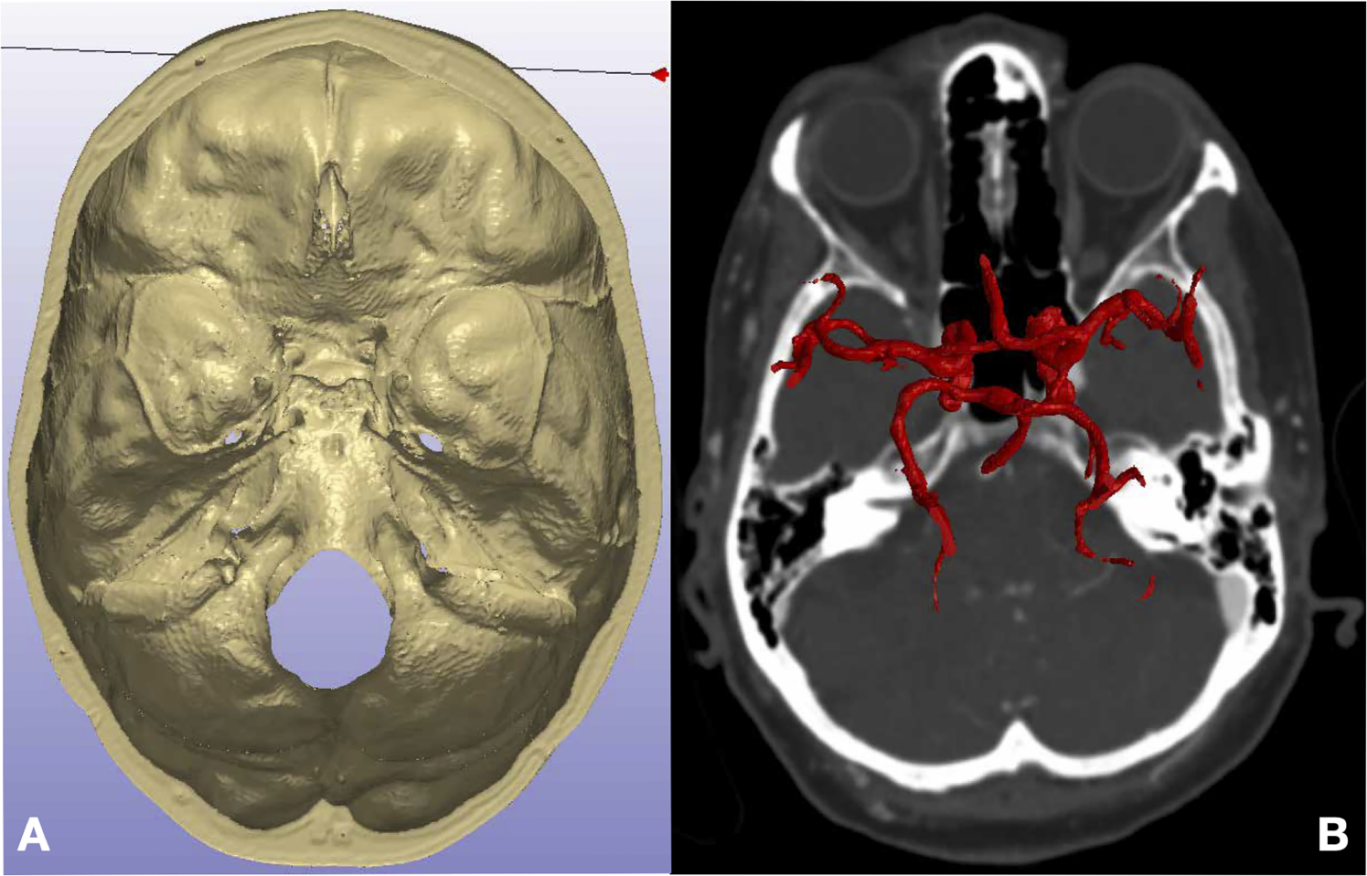
3D Reconstruction: three-dimensional rendering of segmented bone (A) and neurovascular structures (B).

Anonymised DICOM data was uploaded onto a software (Simpleware Scan IP P2019.19*;* www.simpleware.com/software/scanip/*),* which semi-automatically registered and subsequently segmented the image contents into bone, the cochlea, the labyrinth and the neurovasculature (Figure 2). A combination of the landmark and automated registration methods were used within the Simpleware Scan IP software. First, initial manual alignment between scans was achieved by matching landmark pairs between images. The registration was then fine tuned automatically using a grey-scale surface matching algorithm. Segmentation of the five tissue classes was achieved using specific Hounsfield Unit (HU) thresholds, which are outlined in Table 1. Results were reviewed iteratively by the director of anatomy and the head of neurosurgery to ensure segmentation was accurate. The segmented structures were then reconstructed as 3D images (Figure 3) While the brainstem was visible on the CT scan, there was insufficient contrast to achieve accurate segmentation of all it's components. The decision was made to digitally construct it.

**Table 1.** Segmentation thresholds for five tissue classes.

**TISSUE CLASS**	**AVERAGE THRESHOLD (HU)**	**RANGE (HU)**
**Bone**	792	224‐1360
**Cochlea**	‐78	‐310‐154
**Ethmoidal cells**	‐689	‐1000‐378
**Soft tissue anatomy**	61.5	21‐81
**Neurovascular structures**	197.5	104‐291

### Computer Aided Design (CAD)

**Figure 4.**
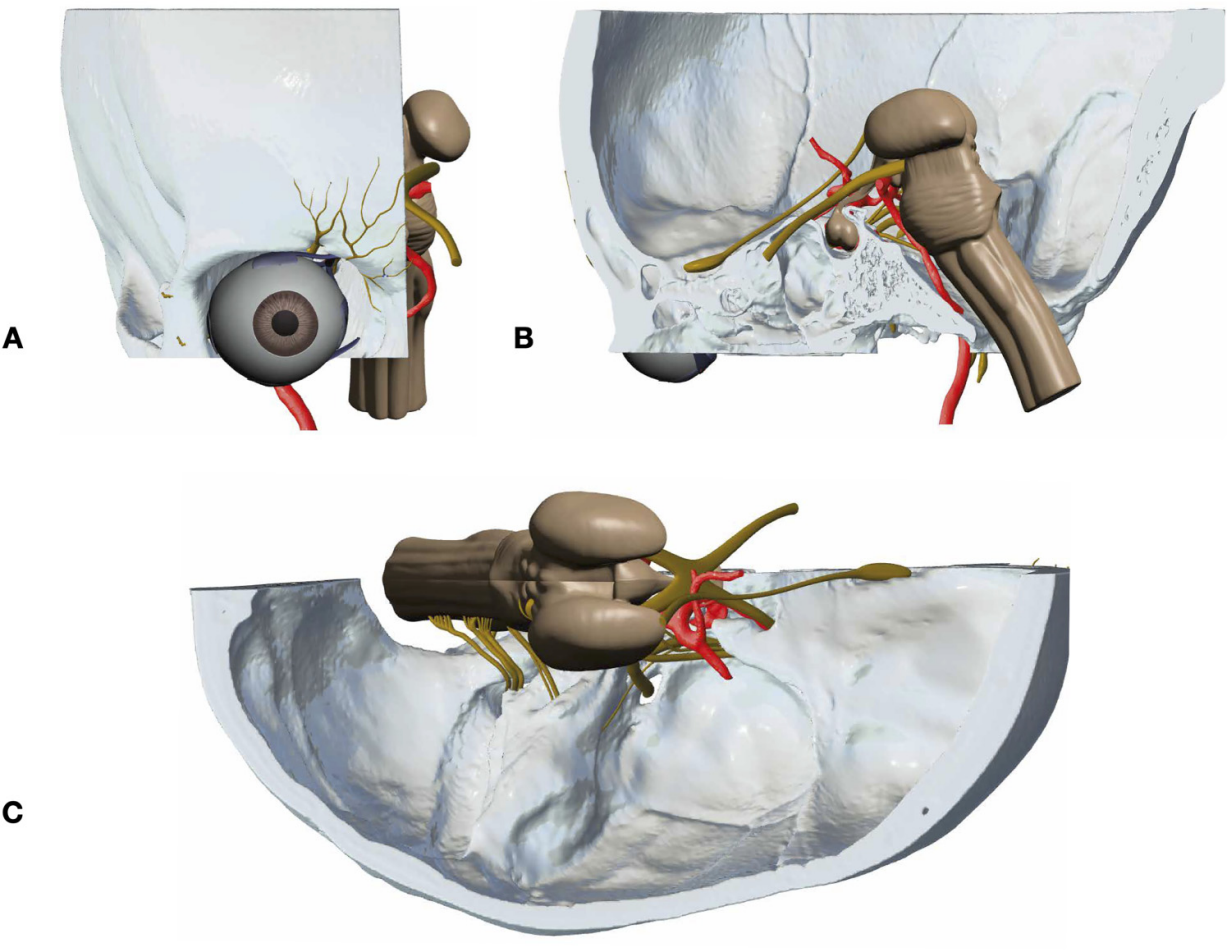
Digital prototype: multiple views of the integrated model prior to printing. anterior, medial and superior views (A, B and C) demonstrate the model components once cranial nerves and brainstem are added to the segmented structures. Close up view in Figure 5 below.

**Figure 5.**
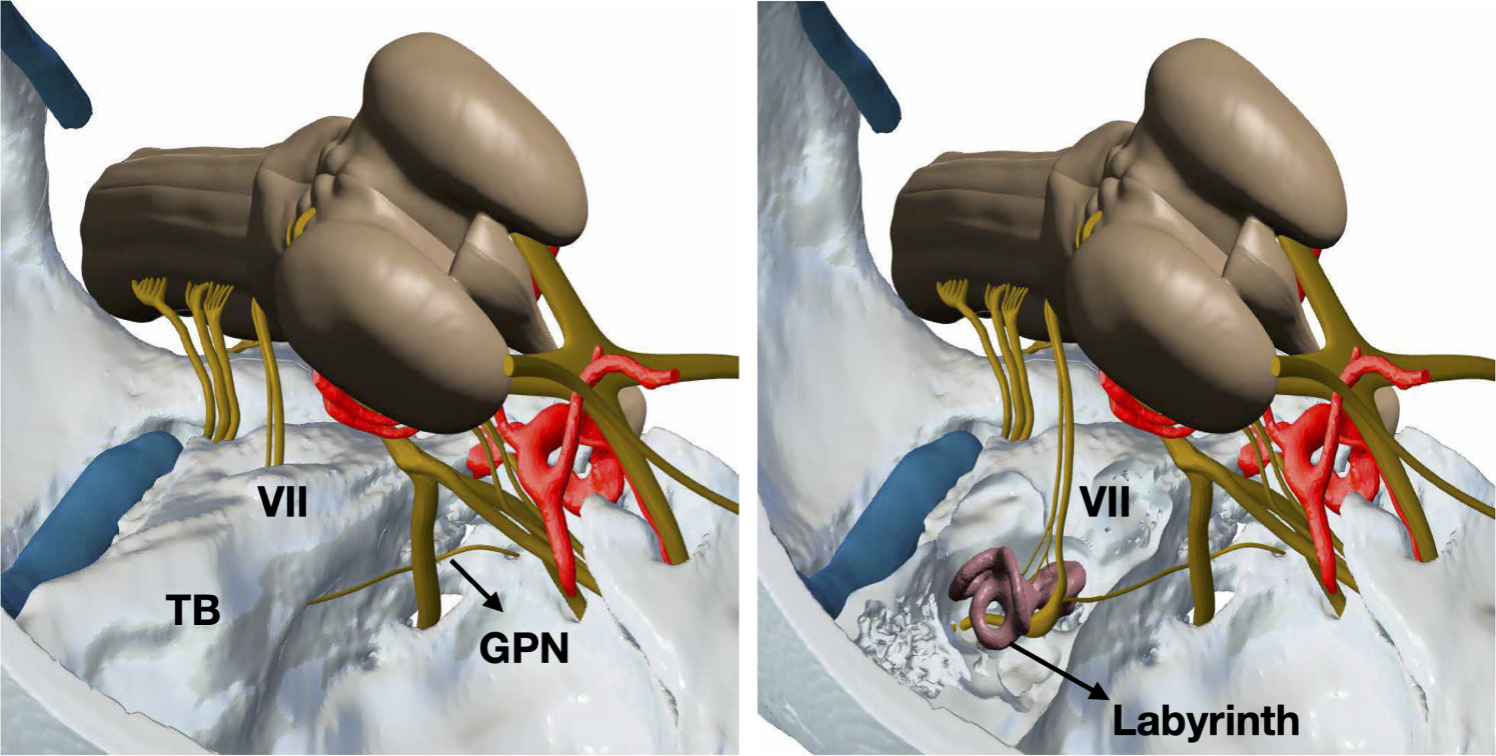
Exposing the temporal bone: The superficial roof of the temporal bone (TB) (left) was sub-segmented to allow for the exposure of the labyrinth and the intratemporal course of the facial nerve (VII). Once printed, the sub-segmented portion will serve as a bony window which can be reversibly removed. GPN = greater petrosal nerve.

The brainstem and cranial nerves were digitally created by expert anatomy artists using *Blender,* an open source 3D software *(v 2.81*, https://www.blender.org). This was conducted under the guidance of the director of anatomy, the lead tutor in neuroanatomy and the neurosurgical clinical team at Oxford University Hospitals. Upon completion, each of the model components was converted to Standard Tessellation Language (.STL) format and rendered as a 3D mesh onto a mesh editing software – *Meshmixer (v 3.5,* http://www.meshmixer.com/download.html*)*. This multipurpose open source software was used to integrate the bony structures, neurovascular supply and soft tissue anatomy into a 3D digital prototype. Once rendered, changes were made to ensure all components were anatomically appropriately aligned. An example of this can be seen in Figure 4. Additionally, it was also used to create a bony window within the temporal bone to visualise the contents of the IAC such as the labyrinth and the intra-temporal course of the facial nerve (Figure 5). The final model contained 5.6 × 10^6^ polygons.

### Material Selection

Materials were selected with three principles in mind:

1. Structures must visually stand out from one another within the skull base and be clearly identifiable. This is essential within the skull base to allow tutors/tutees to appreciate the different structures and their trajectories.
2. Materials should be appropriately durable and haptically representative. In other words, the vascular and neural structures must be flexible and durable, and the skeletal structures rigid and anatomically accurate.
3. Important cranial nerve trajectories must be visible through the bony structures when appropriate. This is particularly important for the facial nerve and its intra-temporal course. This would require for certain segments of bone to be made translucent so as to visualise the nerve trajectory through it. In addition, the accuracy at intricate areas such as the cavernous sinus and orbital apex where cranial nerves intersect were meticulously delineated.

### Printing

In order to fulfil the above principles, we used a 3D printing process known as polymer jetting, or Polyjet 3D printing, which simultaneously processes rigid polymers and elastomeric material to produce functionally graded products.^15^ A Stratasys j750^TM^ printer^16^ was used to support this capability. A combination of Vero ^TM^, which is a rigid polymer, and Tango, an elastomeric polymer, was used to produce a composite anatomical model that was both anatomically accurate as well as suitable for teaching. We scaled the model to 140% of its original size to improve visualisation of small structures.

Computer aided design files were processed by the printer using a printing software for Stratasys printers known as GrabCAD^17^ which additionally calculates the most economical print orientation for the model and scaffolds the model with a removable support polymer where required. Once this was determined the printing commenced. Printing time was 40 hours and the total printing cost was approximately 1300 GBP.

### Post Processing

Prints were cooled for two hours and their support polymers removed by hand. Print components were then washed using sodium hydroxide and metasilicate for five cycles and coated in three layers of flexible acrylic lacquer before being mounted. All twelve cranial nerves were labelled for ease of use for students.

## Results

**Figure 6.**
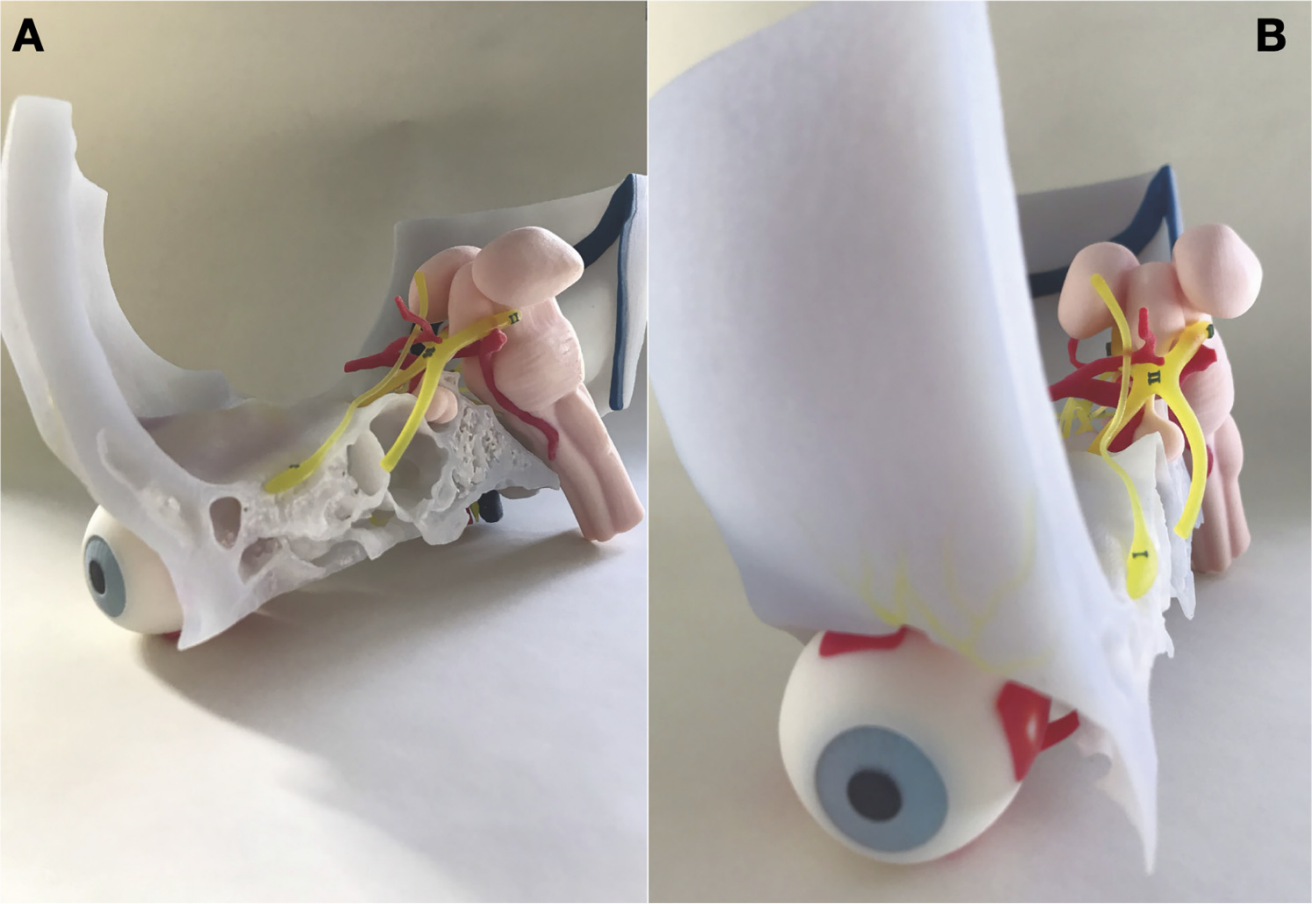
Final model. Printed skull base model viewed from the medial side (A) and anteriorly (B).

**Figure 7.**
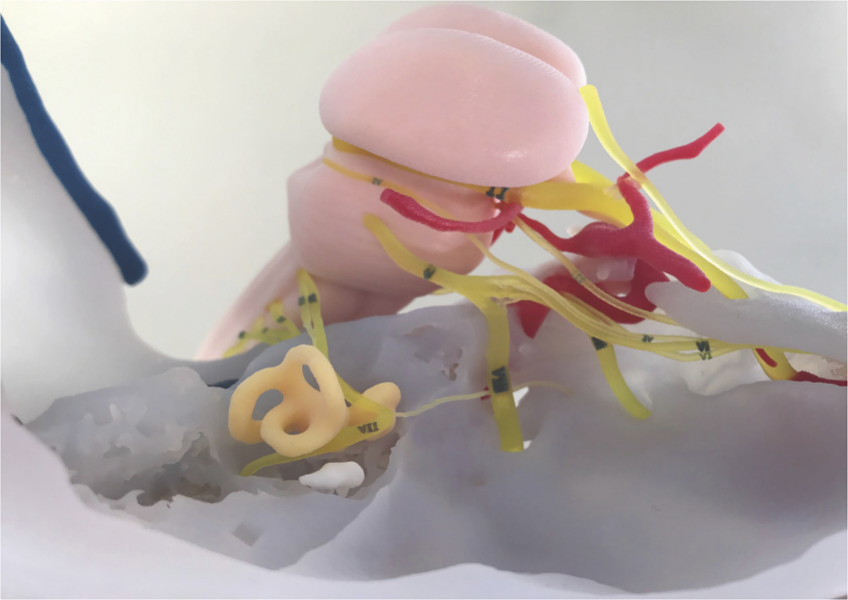
3D Printed model. Final model demonstrating all printed structures as planned in Figures 5 and 6. All cranial nerves and their trajectories are clearly visible, including the trajectory of the facial nerve through the temporal bone window which we had created.

Our model was successfully printed with no complications and can be viewed in Figures 6 and 7. This included accurate representations of the neurovascular contents of the skull base. Notably, we created a bony window, revealing the course of the facial nerve within the temporal bone revealing its relations with the cochlea and vestibular labyrinth.

## Discussion

**Figure 8.**
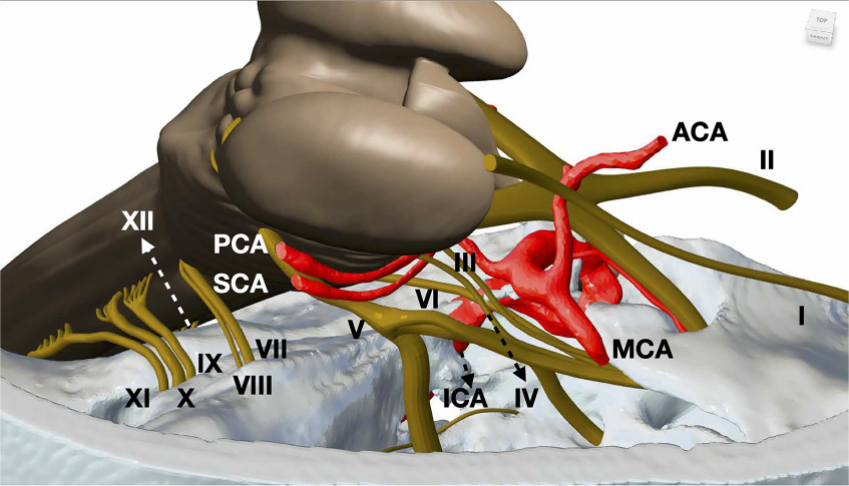
Anatomical relationships within the skull base: A close up view (lateral) demonstrating all 12 cranial nerves (labelled I-XII) alongside the neurovascular structures. Important anatomical relationships were emphasised. (Cranial nerves: I – Olfactory, II – Optic, III – Oculomotor, IV – Trochlear, V – Trigeminal, VI – Abducens, VII – Facial, VIII – Vestibulocochlear, IX – Glossopharyngeal, X – Vagus, XI – Accessory, XII – Hypoglossal. Vascular: ICA - internal carotid artery MCA – middle cerebral artery, ACA – anterior cerebral artery, PCA – posterior cerebral artery, SCA – Superior cerebellar artery).

This is the first 3D printed model of the skull base in our department and will therefor be of much added value to the teaching of trainees and undergraduates. More specifically, it is much more cost effective than cadaveric preparations of the skull base. Our model cost approximately 1300 GBP, took three days from printing to finalising, and requires no storage or maintenance costs. This is significantly more cost-effective compared to cadaveric specimens which can cost up to $2000 to acquire and a further $4000-$6000 to maintain.^18^ Other groups have constructed 3D printed models of the skull base.^19,22^ These are typically limited to the bony structures and have been successfully used both as educational tools as well as for surgical planning. More recently, Lin et al developed three models of the skull-base focussed on endoscopic approaches to surgical resection of tumours of the sella and the acoustic canal.^21^ Due to their focus on endoscopic approaches, these models included a limited but focussed number of internal structures. For example, their sella tumour model only demonstrated cranial nerve II and the internal carotid artery, whereas their acoustic neuroma model only contained cranial nerves VII-XI and the transverse sinus. Comparatively, our single model provides the trajectories of all twelve cranial nerves and their relations to the anterior circulation within the skull base. This provides a more complete model and emphasises clinically relevant relationships between the blood vessels and cranial nerves within the skull base (Figure 8). Additionally, a novel modifiable window was created to visualise the cochlea and the intra-temporal course of the facial nerve. This can aid neurosurgical trainees learning operative middle-fossa or presigmoid approaches in the presurgical planning stage, especially if 3D printed models of prospective patients are used. That said, this work by Lin and colleagues demonstrated some useful features.^22^ For example, rather than utilise a bony window as we did, they used a translucent material throughout, ensuring both the integrity of the bony structures, as well as good visualisation of the surrounding neurovascular structures. This is an adaptation we may consider in our future models.

Our model was developed through a multidisciplinary collaboration between the anatomy department at Oxford University, the Neurosurgical Department at Oxford University Hospitals and an industry partner (3D LifePrints) with an interest in medical applications. As much as possible, we used patient neuroimaging data, although the cranial nerves and brainstem were digitally created by anatomy artists. To ensure appropriate relational anatomy, both the neurosurgical team and director of anatomy at Oxford University regularly reviewed these structures to ensure compatibility with the rest of the model components. Worth noting is that most of the post-processing software used was open source, making this process accessible and available to other educational institutions hoping to replicate our model.

Several lines of evidence support the use of 3D printed models in teaching.^18,19,22^ Chen et al randomised 79 medical students to learning the contents of the skull base using either a 3D printed model, a 2D atlas or a cadaveric model. They demonstrated that students performed best using a 3D printed model as a self-directed learning tool.^19^ A similar study demonstrated superiority of 3D printed models compared to cadaveric models of the vascular supply of the heart.^23^ Based on these findings, we think this model will contribute positively to the education of undergraduate students and surgical trainees at a moderate cost, especially in comparison to the cost of cadaveric courses.^4^ Notably, it is highly reproducible and can be modified to suit several purposes. For example, miniature models can serve as revision tools for students. Alternatively, where resources are scarce or access to cadaveric specimens is limited, 3D printed models can act as a valuable alternative. Finally, patient-specific models can be used as part of pre-operative surgical simulation for trainees or consultants in complex cases.

Notwithstanding the above, our study has some limitations. Our current model relied on two separate patients for the reconstruction of bony surfaces and blood vessels. This required some manipulations of the neurovascular structures to ensure they aligned appropriately with the bony surfaces of the skull base. These changes were minor although ideally, we will aim to use a single patient's imaging for all future reconstructions. Another limitation to be aware of is that development of our temporal bone window required removal of some structures, such as the mastoid air cells. This may theoretically make our model less anatomically realistic, although in practice the benefits of visualising the internal structures outweigh this cost. Finally, we note that some structures were omitted including the posterior circulation neurovascular structures. This was due to the initial focus of the model being aimed at neurosurgical trainees navigating the neurovascular structures within the skull base. That said, as this model is also used for teaching medical students, including the posterior structures may be of benefit for future replications of this model.

In summary, we have developed a framework for developing an anatomically accurate 3D printed model of the skull-base, in a collaborative effort between an academic institution, teaching hospital and an industry partner. This has real potential in adding value to neurosurgical training, where the operative risk can be significant when navigating complex structures through small apertures. Moving forward, we would like to test the use of our model in both undergraduate and specialist surgical education, to assess its utility in light of its cost of production in comparison to other available learning tools. We hope this framework can be accessible for other academic institutions hoping to pilot similar models. We also note its utility for surgical planning and have begun planning models for complex skull base surgery operations, particularly those involving multiple surgical teams in various surgical subspecialties.
